# Right Heart Thrombus in Transit Diagnosed With Focused Cardiac Ultrasound in the Emergency Department

**DOI:** 10.7759/cureus.9354

**Published:** 2020-07-23

**Authors:** Eric J Kalivoda, Kevin Rivera Rodriguez, Gabriel Cabrera

**Affiliations:** 1 Emergency Medicine, Hospital Corporation of America West Florida Graduate Medical Education Consortium/Brandon Regional Hospital, University of South Florida Morsani College of Medicine, Brandon, USA

**Keywords:** focused cardiac ultrasound, thrombus in transit, emergency department, point-of-care-ultrasound, echocardiography, pulmonary embolism (pe), point-of-care ultrasound

## Abstract

The diagnosis of a right heart thrombus (RHTh) in transit associated with pulmonary embolism (PE) is an uncommon phenomenon with a high mortality rate. Timely recognition of RHTh with focused cardiac ultrasound (FOCUS) in cases of suspected PE is necessary for critical decision making in the emergency department (ED). We present a case of RHTh with submassive PE in which the patient underwent successful catheter-directed thrombolysis. This case report illustrates the significance of an emergency physician (EP) implementing FOCUS during the initial ED evaluation to rapidly diagnose RHTh in transit and initiate PE therapy without delay.

## Introduction

Pulmonary embolism (PE) is a potentially fatal entity that is frequently diagnosed in the emergency department (ED) [1,2]. Emergency physicians (EPs) routinely perform focused cardiac ultrasound (FOCUS) to support a prompt ED diagnosis of PE [3-7]. Direct visualization of a right heart thrombus (RHTh) with FOCUS is diagnostic of PE [6]. However, this is rarely encountered in the ED, with the prevalence of RHTh reported to be as low as 4% among PE patients [8]. The coexistence of a RHTh with PE carries an increased mortality risk necessitating aggressive management [8-11]. Previous reports have described the crucial role of EP-performed FOCUS to establish the diagnosis of RHTh [12-18]. We report a case in which EP-performed FOCUS facilitated the expeditious diagnosis and management of RHTh in transit associated with submassive PE.

## Case presentation

A 90-year-old female with a past medical history of hypertension, dyslipidemia, and recent right hip surgery was brought to the ED by Emergency Medical System (EMS) personnel from a nearby rehabilitation facility after experiencing a syncopal event during her physical therapy session. Upon arrival to the ED resuscitation bay, the patient endorsed dyspnea for which EMS had placed her on a non-rebreather mask with high-flow oxygen. There were no reported complaints of chest pain, palpitations, hemoptysis, lower extremity swelling, abdominal pain, flank pain, back pain, headache, vision or speech changes, or focal neurological deficits. 

On initial examination, her vital signs were temperature of 36.4°C, blood pressure 111/59 mmHg, heart rate 112 beats per minute, respiratory rate 34 breaths per minute, and oxygen saturation of 100% on a nasal cannula at 6 L/minute. She was in mild respiratory distress and her lungs were clear to auscultation with symmetric chest rise. Initial electrocardiogram (EKG) revealed sinus tachycardia with premature ventricular complexes. Portable chest radiography demonstrated hyperinflation and cardiomegaly. Intravenous antibiotics and intravenous fluids were ordered after the initial ED evaluation for the empiric treatment of suspected sepsis.

**Figure 1:**
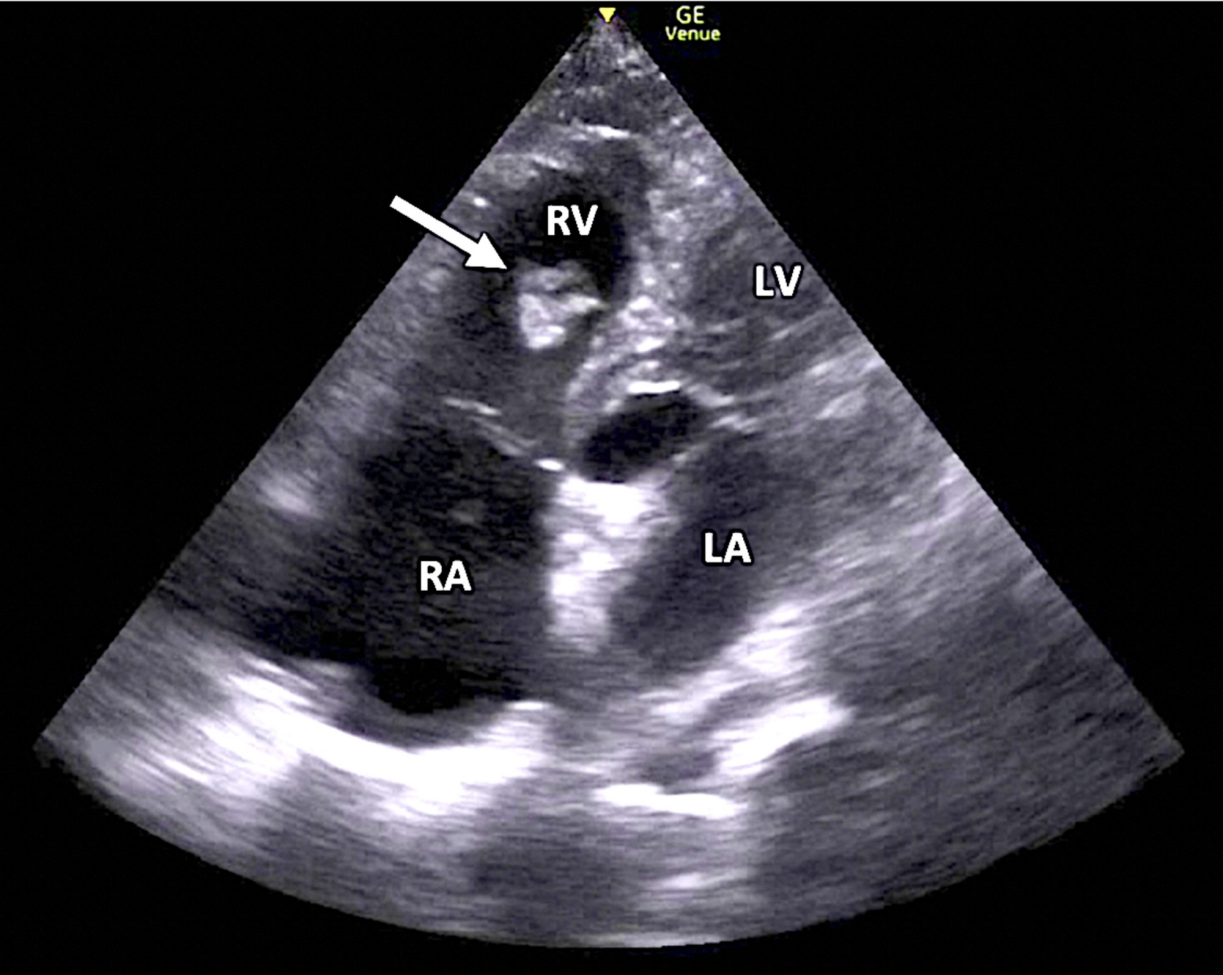
Focused cardiac ultrasound (apical four-chamber window) demonstrating a right heart thrombus (arrow). RV, right ventricle; LV, left ventricle; RA, right atrium; LA, left atrium.

Her initial clinical presentation was suggestive of PE, and FOCUS was immediately performed by an emergency medicine resident physician and ultrasound fellowship-trained ED attending. FOCUS demonstrated a free-floating, mobile echogenic mass located in the right ventricle (RV), consistent with a RHTh in transit en route to the pulmonary vasculature (Figure 1, Video 1). Additional echocardiographic findings of PE, including intraventricular septal flattening, RV dilatation, and diminished tricuspid annular plane systolic excursion (TAPSE), were initially equivocal in the visualized suboptimal cardiac windows. Cardiology was immediately consulted for formal comprehensive echocardiography.

**Video 1:** Focused cardiac ultrasound (apical four-chamber window) demonstrating a right heart thrombus.

**Figure 2:**
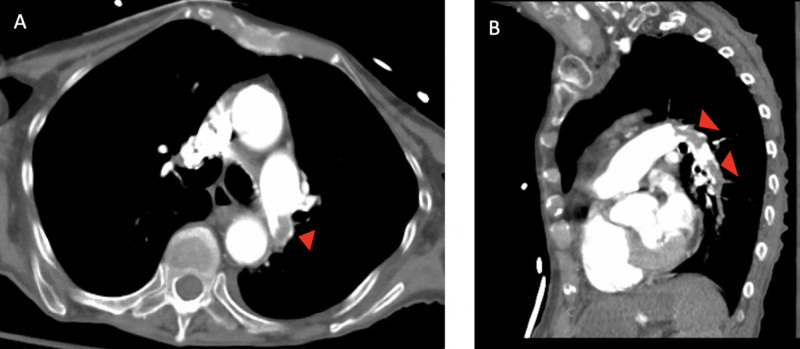
Computed tomography pulmonary angiogram demonstrating an embolus in the left distal pulmonary artery (arrowheads). (A) Axial view. (B) Sagittal view.

Computed tomography pulmonary angiography (CTPA) was obtained expeditiously and confirmed an acute left distal pulmonary artery (PA) PE and bilateral segmental and subsegmental PE (Figure 2). The patient was started on an intravenous heparin infusion. Laboratory results, which arrived following completion of CTPA, were significant for elevated cardiac biomarkers with troponin I of 0.137 ng/mL (0-0.045 ng/mL) and N-terminal pro B-type natriuretic peptide (NT-proBNP) of 1,541 pg/mL (0-450 pg/mL), as well as an initial serum lactate of 9.3 mmol/L (0.4-2.0 mmol/L). 

**Figure 3:**
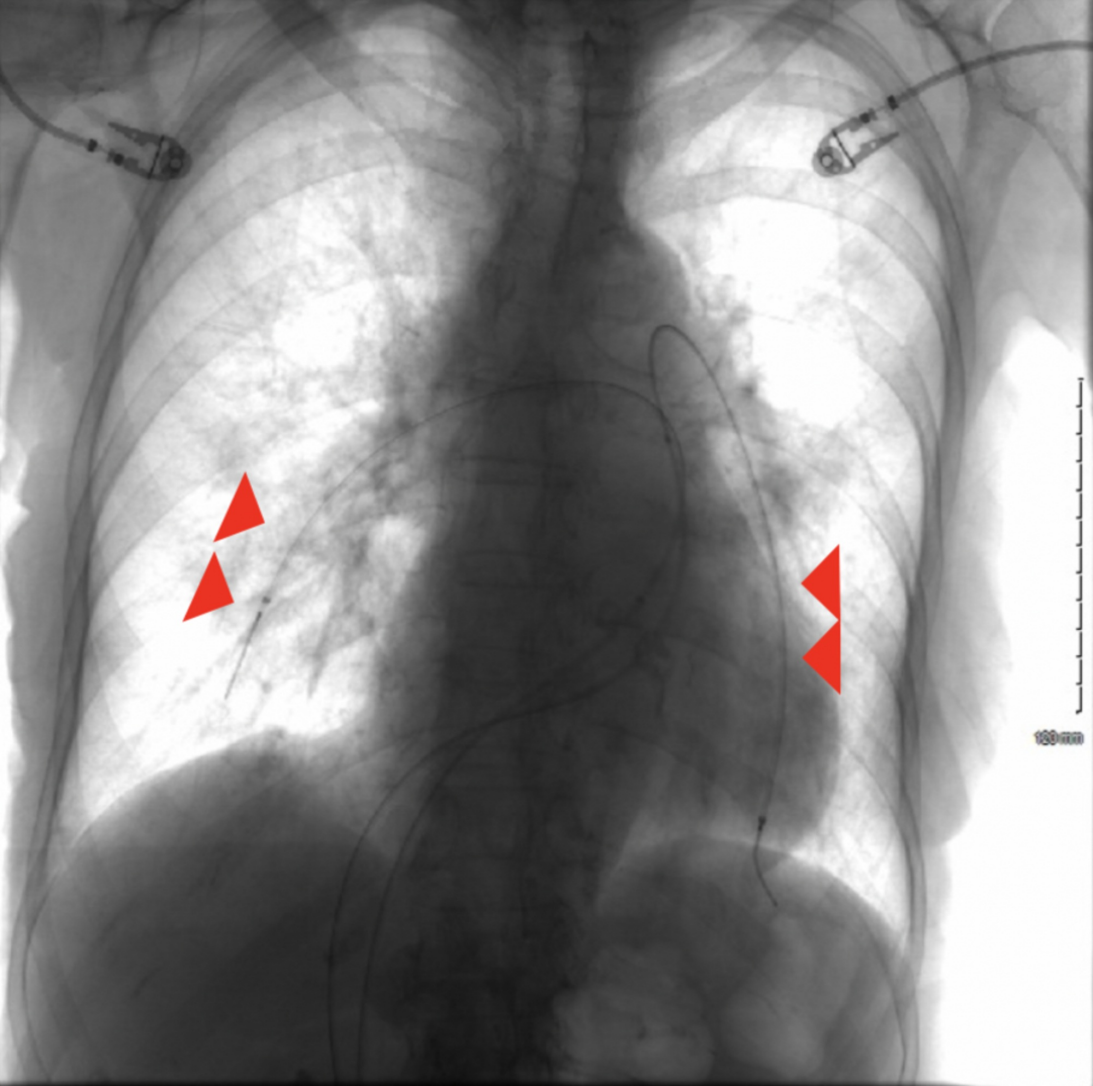
X-ray fluoroscopy demonstrating infusion catheters (arrowheads) placed for thrombolytic administration.

The patient remained hemodynamically stable during the entirety of the ED course. Comprehensive echocardiography later confirmed the presence of a highly mobile RV mass, RV dilatation, reduced RV systolic function, and an estimated peak PA pressure of 43 mmHg. Comprehensive lower extremity ultrasound revealed bilateral occlusive deep venous thrombi (DVT). Interventional radiology (IR) was consulted for catheter-directed thrombolysis (CDT) of submassive PE. The patient was admitted to the intensive care unit (ICU) for further management. IR-performed CDT infusion (0.5 mg/hour alteplase) on the day of admission revealed a mildly elevated PA pressure of 26 mmHg prior to the administration of thrombolytics; re-evaluation by IR the next day demonstrated a normalized PA pressure of 14 mmHg (Figure 3). An inferior vena cava filter was also placed by IR. No complications were reported during her inpatient course due to thrombolytic administration. The patient was ultimately discharged from the hospital with arrangements for hospice care per family request.

## Discussion

FOCUS is an essential bedside tool for the ED diagnosis and risk stratification of PE. EPs have demonstrated that FOCUS integration into the initial ED evaluation of suspected PE has invaluable benefits in establishing support for the diagnosis before confirmatory CTPA studies [3-7]. Risk stratification of PE involves a global assessment of four factors: (1) presence of echocardiographic signs of RV dysfunction (RVD), including but not exclusive to interventricular septal wall flattening, RV dilatation, and decreased TAPSE; (2) elevations of cardiac biomarkers (troponins and natriuretic peptides); (3) hemodynamic status of shock/hypotension; (4) pulmonary embolism severity index (PESI) [19]. In this report, the patient notably had an initial equivocal FOCUS assessment for the common echocardiographic findings of PE upon ED arrival, due in some degree to technically challenging cardiac windows. However, the definitive diagnosis of PE by visualization of a RHTh by EP-performed FOCUS, as is described in this case, is a notably rare occurrence [8,12-18]. Patients with PE and RHTh have been shown to have a significantly shorter duration of symptoms, which is consistent with our suspicions that our patient’s PE presentation was hyperacute and rapidly evolving as thrombi from the RHTh entered the pulmonary circulation [8]. The patient likely precipitously developed echocardiographic RVD during the ED course, as the RV death spiral phenomenon worsened with progressive pulmonary obstruction [2]. Despite maintaining hemodynamic stability, the patient was ultimately classified as intermediate-high risk for early PE mortality, given that the comprehensive echocardiogram demonstrated signs of RVD (in addition to FOCUS identification of RHTh), elevations of both cardiac biomarkers, and a PESI class V (very high 30-day mortality risk). The described case highlights the importance of EPs incorporating FOCUS into clinical practice for PE risk stratification. 

The early recognition of RHTh and associated PE with FOCUS is vital to guide clinical management and achieve a favorable clinical outcome, especially given the in-hospital mortality rate for patients with a RHTh in transit has been described to be approaching 45% [1,2,8-11]. Previous studies of RHTh cases have suggested a mortality benefit from thrombolysis versus anticoagulation alone [11]. The management of intermediate-risk (submassive) PE with CDT has indicated promising short-term benefits as a therapeutic strategy [20]. Our patient case describes successful CDT for the management of submassive PE with RHTh. This case highlights the critical role of bedside FOCUS in assisting EPs to direct prompt and appropriate PE therapy. 

## Conclusions

A RHTh in transit coexisting with a PE is a disease process that is often fatal if not recognized. FOCUS performed early during the ED encounter can facilitate EPs to make this diagnosis definitively and initiate timely treatment. Outcomes-based research of EP-performed FOCUS combined with newer therapeutic strategies for submassive PE (with and without the presence of RHTh) warrant further investigation.
